# Validation of a novel neuroimaging signature for dementia and clinical Alzheimer's disease in the population-based Rotterdam study

**DOI:** 10.1177/13872877251315044

**Published:** 2025-02-16

**Authors:** Jacqueline J Claus, Mathijs T Rosbergen, Gennady V Roshchupkin, M Arfan Ikram, Meike W Vernooij, Frank J Wolters

**Affiliations:** 1 Department of Epidemiology, Erasmus MC University Medical Center, Rotterdam, The Netherlands; 2 Department of Radiology & Nuclear Medicine and Alzheimer Center Erasmus MC, Erasmus MC University Medical Center, Rotterdam, The Netherlands; 3 Department of Medical Informatics, Erasmus MC University Medical Center, Rotterdam, The Netherlands

**Keywords:** Alzheimer's disease, cortical thickness, dementia, hippocampus, magnetic resonance imaging

## Abstract

**Background:**

A novel neuroimaging signature of regional cortical thickness on brain MRI recently showed high potential for Alzheimer's disease and related dementias (ADRD) risk stratification in the community. How these findings translate to other populations, remains undetermined.

**Objective:**

We aimed to replicate this novel ADRD neuroimaging marker in the population-based Rotterdam Study.

**Methods:**

We included all participants from the population-based Rotterdam Study with brain-MRI between 2005–2016, and derived the signature using FreeSurfer. We computed hazard ratios and C-statistics for 10-year dementia risk, and betas for cross-sectional associations with cognition, comparing the novel signature to hippocampal volume, mean cortical thickness, and another cortical thickness signature (Dickerson's).

**Results:**

Of 3249 participants (mean age 71.3 ± 8.0 years), 294 developed dementia (74.8% clinical AD) during a mean follow-up of 8.1 years. The novel ADRD signature had similar magnitude of associations as Dickerson's signature and cortical thickness for AD dementia (HR per 1-SD increase 0.87;0.78–0.96), but performed worse than all markers for all-cause dementia. Of the four neuroimaging markers, hippocampal volume showed the strongest associations with both risk of all-cause dementia and clinical AD dementia. The ADRD had the weakest association with general cognitive function (β per 1-SD increase 0.04;0.02–0.06), and executive function (β per 1-SD increase 0.02;0.00–0.04), followed by cortical thickness and Dickerson's, and hippocampal volume showed the strongest associations.

**Conclusions:**

In this community-based study, the novel cortical thickness signature did not outperform hippocampal volume for dementia risk stratification. The importance of replication studies underlines the value of the current study. Replicating research findings is essential to establish robust biomarkers for dementia risk prediction.

## Introduction

Dementia is among the largest health problems, with around 57 million people currently living with dementia worldwide.^1^ With no current treatment available, early risk stratification could enable targeted prevention and assist in personalized selection for treatment in clinical trials. Brain magnetic resonance imaging (MRI) is a non-invasive investigative tool that can relatively easily be applied to large populations at risk of dementia for risk stratification. While the body of literature on novel brain MRI biomarkers for dementia risk is growing, few are replicated and translation to routine clinical practice remains challenging.^2^

Recently, a novel neuroimaging signature for Alzheimer's disease and related dementias (ADRD) was identified from cortical thickness maps in the Framingham Heart Study (FHS).^3^ This signature showed high risk estimates for all-cause dementia and AD dementia, that went above and beyond established MRI biomarkers including hippocampal volume, mean cortical thickness and cortical thickness in Dickerson's signature.^4^ The investigators replicated analyses in the same report using data of the University of California Davis Alzheimer's Disease Research Center (UCD-ADRC) cohort, with similar results.^3^ However, it remains undetermined how these findings translate to other populations across the globe.

We therefore aimed to determine the association between the novel cortical thickness neuroimaging biomarker and cognition and dementia risk in the population-based Rotterdam Study.

## Methods

### Study design

The Rotterdam Study is an ongoing prospective population-based cohort study investigating determinants and occurrence of disease in persons aged 40 years and older. The study started in 1990 and now comprises 17,931 individuals living in the Ommoord suburb of Rotterdam, the Netherlands. The design of the Rotterdam Study has been described in detail previously.^5^ In brief, participants are invited for interview and extensive in-person examination at a dedicated research center every 3–6 years. For the current study, we included all participants who underwent brain MRI between 2005 and 2016 (N = 5862), of whom 5586 had valid measurement of either hippocampal volume, overall mean cortical thickness or mean cortical thickness in Dickerson's or the ADRD signature. We excluded individuals with previous stroke (n = 180), insufficient information on dementia at time of first scan (n = 68) or prevalent dementia at time of MRI (n = 57). For analyses of dementia risk, we included only participants over the age of 60 (n = 3254/5586). For cross-sectional analyses of cognition, we excluded 221/5586 (4.0%) participants who did not undergo cognitive assessment.

### Ethics approval

The Rotterdam Study has been approved by the Medical Ethics Committee of the Erasmus MC and by the Ministry of Health, Welfare and Sport of the Netherlands, implementing the Population Screening Act: Rotterdam Study.

### Brain MRI acquisition and processing

All participants underwent scanning on a 1.5-T MRI device (GE Healthcare) using a multisequence protocol consisting of T1-weighted, proton density-weighted, fluid-attenuated inversion recovery, and T2-weighted sequences. For brain volumetry, T1-weighted (voxel size 0.49 × 0.49 × 1.6 mm^3^), proton density–weighted (voxel size 0.6 × 0.98 × 1.6 mm^3^), and the fluid-attenuated inversion recovery (FLAIR) (voxel size 0.78 × 1.12 × 2.5 mm^3^) scans were used for automated segmentation of supratentorial gray matter, white matter, cerebrospinal fluid (CSF), and white matter hyperintensities, using an in-house segmentation method.^6,7^ All segmentations were visually inspected, and manually corrected if needed. Total brain volume was the sum of gray matter, normal-appearing white matter, and white matter hyperintensity volume. Supratentorial intracranial volume was estimated by summing gray and white matter (consisting of the sum of normal-appearing white matter and white matter lesion volume) and CSF volumes.^7^ Additionally, all T1-weighted MR images were processed using FreeSurfer (version 6.0) to obtain hippocampal volume^8^ and cortical thickness measurements. Hippocampal volume was defined as the sum of the left and the right hippocampal volumes. Total brain volume was defined as the sum of white and gray matter as a percentage of the total intracranial volume. We used the mean cortical thickness and the surface area-weighted average of the AD cortical thinning signature defined by Dickerson and colleagues.^4^ This signature consists of multiple regions, including the entorhinal cortex, parahippocampus, inferior parietal lobe, pars opercularis, pars orbitalis, pars triangularis, inferior temporal, temporal pole, precuneus, supramarginal gyrus, superior parietal and superior frontal regions.

### ADRD signature

**Figure 1.**
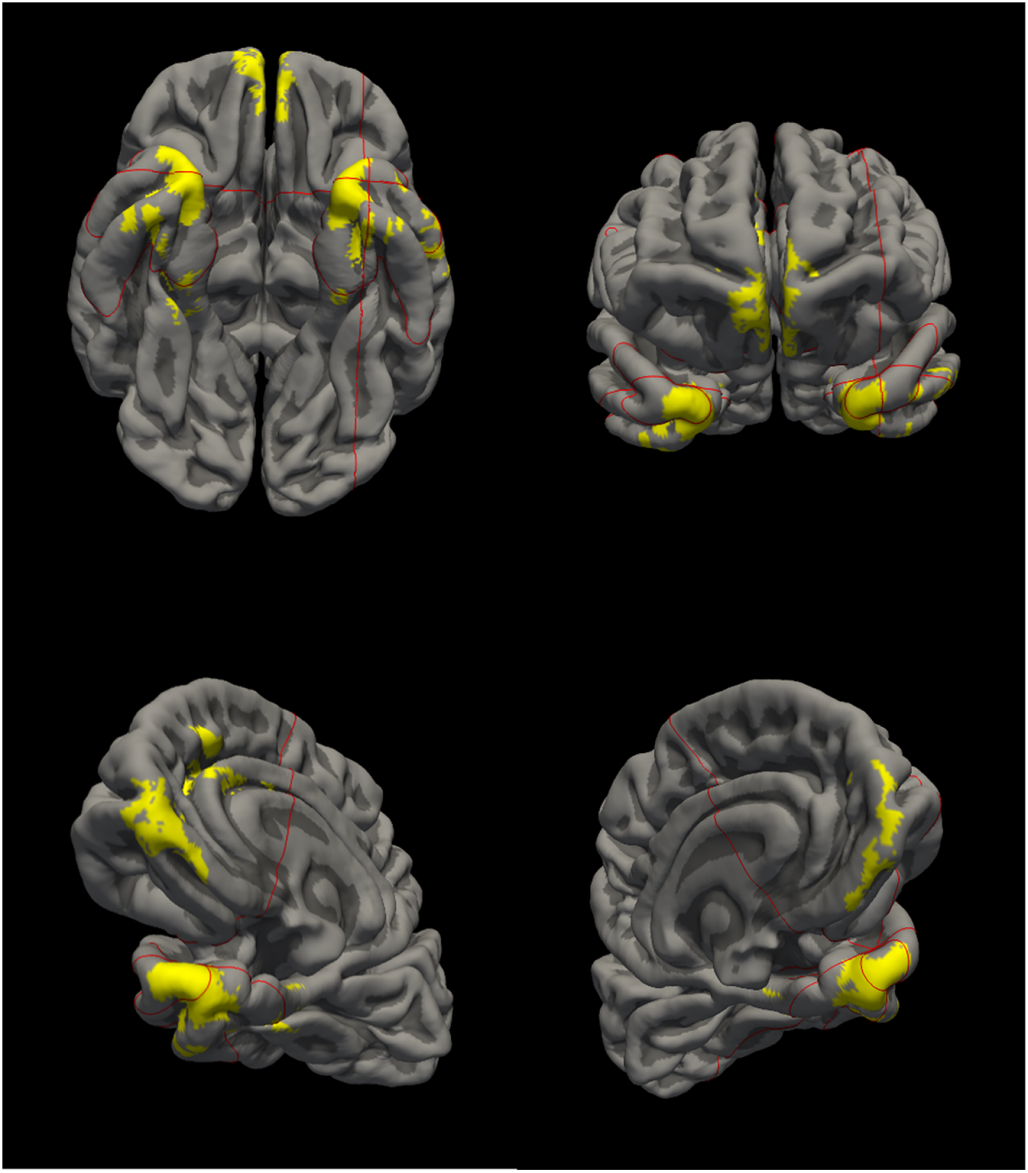
Visualization of the ADRD signature.

Satizabal and colleagues previously derived an ADRD signature from cortical thickness maps using brain MRI of participants with AD dementia and matched controls from the Framingham Heart Study.^3^ They determined the cortical thickness in MRI scans using the DiReCT diffeomorphism-based application.^9^ Further details on the development of the ADRD signature have been described previously.^3^ For the current study, we have used the region of interest (ROI) of the signature in MNI space as provided by Satizabal and colleagues (private correspondence). To determine the mean cortical thickness within this ROI, we registered the ROI from MNI space to FreeSurfer space, and processed Rotterdam Study imaging using FreeSurfer 6.0 to determine for each study participant mean cortical thickness within the ADRD signature ROI. The ROI in FreeSurfer space is shown in Figure 1.

ADRD signature ROI projected on white matter volume in 3D in the population-based Rotterdam study.

ADRD: Alzheimer's disease related dementias; ROI: region of interest.

### Dementia ascertainment

Participants were screened for dementia at baseline and every 3–6 years during follow-up examinations using the Mini-Mental State Examination (MMSE) and the Geriatric Mental Schedule (GMS) organic level. Those with an MMSE score of <26 or a GMS organic level score of >0 were further examined using the Cambridge Examination for Mental Disorders in the Elderly diagnostic interview. Additionally, participants were continuously under surveillance for dementia through the electronic linkage between the study database and medical records from general practitioners and the Regional Institute of Outpatients Mental Health Care. The general practitioner functions as a gatekeeper within the Dutch healthcare system, receiving written information of any medical specialists’ consultations of their patients. The final diagnosis of dementia and its most common subtypes was made by a consensus panel led by a neurologist based on the standard criteria for all-cause dementia (DSM-III-R) and clinical Alzheimer's disease (NINCDS-ADRDA).

### Cognitive assessments

Assessment of cognitive function was routinely performed at center visits through a cognitive test battery, including the Stroop test,^10^ measuring processing and attention, the Word Fluency test (WFT),^11^ measuring efficiency of searching long term memory, the Letter Digit Substitution test (LDST),^12^ measuring processing speed and executive function, 15-word verbal learning test (15-WLT),^13^ measuring verbal learning and memory, and Purdue Pegboard test (PPB),^14^ measuring dexterity and fine motor speed. In this study, we derived a measure of global cognition (g-factor) from principle component analysis of available test scores.^15^ The g-factor is defined as the first unrotated component of the principle component analysis. A higher g-factor indicates a better cognitive performance. Executive function was defined as the mean of standardized values of the Stroop test, the WFT and the LDST (weighted half). Episodic memory was defined as the score from the 15-WLT.

### Assessment of APOE genotype

Apolipoprotein E *(APOE*) genotype was determined using polymerase chain reaction on coded DNA samples or using biallelic TaqMan assays (TaqMan Gene Expression Assays; Thermo Fisher Scientific, Waltham, MA) (rs7412 and rs429358).^16,17^ *APOE*-ε4 carriers were defined as carrying at least one ε4 allele (ε3/ε4, ε2/ε4 and ε4/ε4).

### Statistical analyses

Missing data on cognitive testing was imputed using 5-fold multiple imputation when data of at least one cognitive test of the test battery was available, based on age, sex, education, MMSE and other available cognitive tests. Imaging markers included mean cortical thickness within the ADRD signature and Dickerson's signature, overall mean cortical thickness (averaged over the entire cortex) and hippocampal volume. In line with the previous report by Satizabal et al.,.^3^ we modelled all markers as continuous measures (for hazard ratios [HR] with 95% confidence intervals [CI] per 10 units increase), and comparing the bottom quartile to the upper three quartiles. To enhance comparability between measures, we additionally standardized measures of each imaging marker to provide HRs with 95% CIs per standard deviation (SD) increase of each imaging marker.

Follow-up time was measured in years from the date of the MRI examination until dementia diagnosis, death, loss-to-follow-up, or censoring at 10 years of follow-up, whichever came first (in line with the report by Satizabal et al.).^3^ We performed Cox proportional hazard models to compute HRs for each imaging marker across the 10-year incidence of all-cause and AD dementia. All models were adjusted for age and sex, and additional models also for other brain MRI markers of dementia (total brain volume, hippocampal volume, and white matter hyperintensities as percentage of intracranial volume). The C-statistic was computed to quantify the predictive discrimination.

Next, we determined the cross-sectional association of imaging markers with cognitive function (episodic memory, executive function and global cognition), using linear regression.

We stratified all analyses by sex and tested for multiplicative interactions between the ADRD signature and sex in the Cox regression models, and additive interaction in the linear regression models for cognition. Last, we performed linear regression models to relate *APOE*-ε4 carrier status to the ADRD signature.

All analyses were performed in R (version 4.2.1; packages: tidyverse, dplyr, lubridate, here, rio, survival).

## Results

### Participants characteristics

We included 5060 participants for analyses on cognition (mean age 64.7 ± 10.8 years, 55.6% women) and 3249 for analyses on dementia risk (mean age 71.3 ± 8.0 years, 55.8% women) (Table 1).

**Table 1.** Participants’ characteristics.

	Cognition sample (N = 5060)	Dementia sample (N = 3249)
Age, mean (±SD)	64.7 (10.8)	71.3 (8.0)
Women, n (%)	2813 (55.6)	1814 (55.8)
Education, n (%)		
Lower	444 (8.8)	307 (9.4)
Intermediate	3452 (68.2)	2312 (71.2)
Higher	1131 (22.4)	585 (18.0)
*APOE* ε4 carriers, n (%)	1426 (28.2)	836 (26.5)
MRI markers, mean (SD)		
ADRD signature ROI, mm	2.93 (0.14)	2.92 (0.14)
Dickerson's signature, mm	2.49 (0.10)	2.46 (0.10)
Cortical thickness, mm	2.45 (0.09)	2.42 (0.09)
Hippocampal volume, cm^3^	7.78 (0.85)	7.56 (0.84)
Total brain volume, cm^3^	942.09 (105.28)	920.86 (103.41)
WMH volume, cm^3^, median (IQR)	3.04 (4.70)	4.56 (7.17)
Cognitive measures*, mean (SD)		
Executive function	0.03 (0.78)	−0.04 (0.50)
Episodic memory	0.03 (0.99)	−0.18 (0.95)
General cognition	0.04 (0.98)	−0.32 (0.95)

AD: Alzheimer's disease; SD: standard deviation; ADRD: Alzheimer's disease related dementias; *APOE*: apolipoprotein E; MRI: magnetic resonance imaging; ROI: region of interest; WMH: white matter hyperintensities; IQR: interquartile range. *Represents standardized values.

Among the neuroimaging markers, overall cortical thickness and Dickerson's cortical thickness showed a high correlation (Supplemental Figure 1). In contrast, hippocampal volume demonstrated the weakest correlation with the other three measures.

### Incident dementia

Of 3249 participants, 303 developed dementia within 10 years from baseline, during a mean follow-up of 8.7 (±2.1) years. Follow-up for incident dementia was virtually complete (96.0% of potential person-years). Increased cortical thickness in the ADRD signature was associated with lower incidence of all-cause dementia (HR [95%CI] per 1-SD increase: 0.89 [0.90–1.00]) and clinical AD dementia (HR: 0.83 [0.72–0.95]; Table 2). Compared to individuals in the upper three quartiles, those in the lowest quartile of the ADRD signature had higher risk of both all-cause dementia and clinical AD dementia in models adjusting for age and sex (Table 2). However, risk estimates for the ADRD signature were similar or lower than those for mean cortical thickness, the Dickerson's signature, and hippocampal volume (Table 2).

**Table 2.** Head-to-head comparison of neuroimaging markers of 10-year dementia risk in the Rotterdam study.

		ADRD signature	Dickerson's	Cortical thickness	Hippocampal volume
All-cause dementia					
Dementia N/Total N		303/3249	303/3249	303/3249	303/3249
Model 1	Continuous: *HR (95% CI)*	0.86 (0.80–0.93)	0.75 (0.66–0.85)	0.73 (0.64–0.83)	0.94 (0.93–0.96)
	*C-statistic*	0.79	0.80	0.80	0.80
	Standardized: *HR (95%CI)*	0.82 (0.73–0.91)	0.75 (0.66–0.85)	0.76 (0.67–0.85)	0.61 (0.52–0.71)
	*C-statistic*	0.79	0.80	0.80	0.80
	Q1 versus Q2–4: *HR (95% CI)*	1.39 (1.10–1.76)	1.89 (1.47–2.42)	1.89 (1.48–2.42)	1.66 (1.27–2.17)
	*C-statistic*	0.79	0.80	0.80	0.80
Model 2	Continuous: *HR (95% CI)*	0.92 (0.85–1.00)	0.81 (0.71–0.92)	0.79 (0.69–0.91)	0.95 (0.93–0.96)
	*C-statistic*	0.81	0.81	0.81	0.81
	Standardized: *HR (95% CI)*	0.89 (0.80–1.00)	0.81 (0.71–0.92)	0.82 (0.72–0.92)	0.62 (0.53–0.72)
	*C-statistic*	0.81	0.81	0.81	0.81
	Q1 versus Q2–4: *HR (95% CI)*	1.21 (0.96–1.54)	1.69 (1.31–2.18)	1.67 (1.30–2.14)	1.63 (1.24–2.13)
	*C-statistic*	0.81	0.81	0.81	0.80
Clinical AD dementia					
AD N/Total N		215/3249	215/3249	215/3249	215/3249
Model 1	Continuous: *HR (95% CI)*	0.81 (0.73–0.90)	0.73 (0.63–0.86)	0.73 (0.62–0.87)	0.94 (0.92–0.96)
	*C-statistic*	0.81	0.81	0.81	0.82
	Standardized: *HR (95% CI)*	0.75 (0.66–0.86)	0.74 (0.63–0.86)	0.76 (0.65–0.88)	0.60 (0.50–0.73)
	*C-statistic*	0.81	0.81	0.81	0.82
	Q1 versus Q2–4: *HR (95% CI)*	1.82 (1.36–2.44)	1.89 (1.38–2.59)	1.81 (1.33–2.47)	1.81 (1.29–2.56)
	*C-statistic*	0.81	0.81	0.81	0.81
Model 2	Continuous: *HR (95% CI)*	0.87 (0.78–0.96)	0.80 (0.68–0.94)	0.80 (0.68–0.96)	0.94 (0.92–0.97)
	*C-statistic*	0.82	0.82	0.82	0.82
	Standardized: *HR (95% CI)*	0.83 (0.72–0.95)	0.80 (0.69–0.94)	0.83 (0.71–0.96)	0.61 (0.51–0.74)
	*C-statistic*	0.82	0.82	0.82	0.82
	Q1 versus Q2–4: *HR (95% CI)*	1.58 (1.17–2.12)	1.67 (1.21–2.30)	1.57 (1.14–2.15)	1.78 (1.26–2.51)
	*C-statistic*	0.82	0.82	0.82	0.82

AD: Alzheimer's disease; ADRD: Alzheimer's disease related dementias; HR: hazard ratio; CI: confidence interval; C-statistic: concordance statistic. Continuous represents hazard ratios per tenth point of the imaging marker. Standardized represents hazard ratios per standard deviation increase of the imaging marker.

Model 1: adjusted for age and sex

Model 2: adjusted for age, sex, and MRI markers (i.e., total brain volume, hippocampal volume [except hippocampal signature], white matter hyperintensities)

After further adjustment for total brain volume, hippocampal volume and volume of WMH, estimates attenuated for all measures of cortical thickness, driven by effects of hippocampal volume. Consequently, the risk estimate of the ADRD signature in the quartile analysis was no longer statistically significant (HR: 1.21 [95% CI: 0.96–1.54]) and lower than for the other cortical thickness measures (Table 2). For clinical AD dementia, the categorized ADRD signature showed similar performance compared to Dickerson's and cortical thickness, although risk was still highest for individuals in the lowest quartile of hippocampal volume. Discriminatory value, as measured by the C-static, was similar across imaging markers (Table 2), due in large part to a large contributing value of age to these models.

Risk estimates for the lowest quartile of the ADRD signature tended to be higher for women than for men, both for all-cause dementia (women: HR 1.53 [1.14–2.05]; and men: HR 1.14 [0.79–1.74]) and clinical AD dementia (women: HR 1.97 [1.39–2.79]; and men: HR: 1.53 [0.90–2.60]), but neither interaction was statistically significant (p_interaction _= 0.15 and 0.11, respectively).

### Cognitive outcomes

Increased thickness in the ADRD signature cortical was associated with better general cognitive function (per 1-SD increase β: 0.04; 95% CI: 0.02–0.06), executive function (per 1-SD increase β: 0.02; 95% CI: 0.00–0.04), and episodic memory (per 1-SD increase β: 0.07; 95% CI: 0.05–0.10) (Table 3). In line with the results on dementia, hippocampal volume showed the strongest association with general cognition, followed by cortical thickness, Dickerson's signature, and the ADRD signature (Table 3). Results were similar in the quartile analyses of all imaging markers. Lower quartiles of hippocampal volume (β −0.27; 95% CI: −0.32–-0.22), Dickerson's (β: −0.18; 95%CI: −0.23–-0.14) and cortical thickness (β: −0.18; 95%CI: −0.23–-0.13) were associated with lower standardized values of executive function, but only marginally for the lowest quartiles of the ADRD signature (−0.04; −0.09–0.00). Only for episodic memory, the lowest quartile of the ADRD signature showed the strongest association of all four markers (β: −0.13; −0.19–-0.07), but this difference was not observed in the continuous analyses per standard deviation (Table 3).

**Table3.** Head-to-head comparison of neuroimaging markers of cognitive test performance in the Rotterdam study.

		ADRD signature	Dickerson's	Cortical thickness	Hippocampal volume
Episodic memory*					
Model 1	Continuous: β	0.05 (0.03–0.07)	0.07 (0.04–0.10)	0.08 (0.04–0.11)	0.01 (0.00–0.01)
	Standardized: β	0.07 (0.05–0.10)	0.07 (0.04–0.10)	0.07 (0.04–0.10)	0.04 (0.01–0.08)
	Q1 versus Q2–4: β	−0.13 (−0.19–−0.07)	−0.07 (−0.14–−0.01)	−0.10 (−0.17–−0.04)	−0.07 (−0.14–0.00)
Model 2	Continuous: β	0.04 (0.02–0.06)	0.06 (0.02–0.09)	0.06 (0.03–0.10)	0.00 (0.00–0.01)
	Standardized: β	0.06 (0.03–0.08)	0.06 (0.02–0.09)	0.06 (0.03–0.09)	0.04 (0.01–0.07)
	Q1 versus Q2–4: β	−0.10 (−0.16–−0.04)	−0.05 (−0.12–0.01)	−0.08 (−0.15–−0.02)	−0.07 (−0.13–0.00)
Executive function*					
Model 1	Continuous: β	0.02 (0.00–0.03)	0.08 (0.06–0.10)	0.10 (0.07–0.12)	0.02 (0.01–0.02)
	Standardized: β	0.02 (0.00–0.04)	0.08 (0.06–0.10)	0.09 (0.06–0.11)	0.13 (0.11–0.16)
	Q1 versus Q2–4: β	−0.04 (−0.09–0.00)	−0.18 (−0.23–−0.13)	−0.18 (−0.23–−0.14)	−0.27 (−0.32–−0.22)
Model 2	Continuous: β	0.01 (0.00–0.03)	0.07 (0.05–0.09)	0.09 (0.06–0.11)	0.01 (0.01–0.02)
	Standardized: β	0.02 (0.00–0.04)	0.07 (0.05–0.09)	0.08 (0.05–0.10)	0.12 (0.10–0.15)
	Q1 versus Q2–4: β	−0.03 (−0.08–0.01)	−0.16 (−0.20–−0.11)	−0.16 (−0.21–−0.11)	−0.25 (−0.30–−0.20)
General cognitive function*					
Model 1	Continuous: β	0.03 (0.01–0.05)	0.10 (0.08–0.13)	0.12 (0.09–0.15)	0.02 (0.01–0.02)
	Standardized: β	0.04 (0.02–0.06)	0.10 (0.08–0.13)	0.11 (0.08–0.13)	0.14 (0.12–0.17)
	Q1 versus Q2–4: β	−0.06 (−0.11–−0.01)	−0.20 (0.26–−0.14)	−0.23 (−0.28–−0.17)	−0.28 (−0.34–−0.23)
Model 2	Continuous: β	0.02 (0.00–0.04)	0.09 (0.06–0.11)	0.10 (0.07–0.13)	0.02 (0.01–0.02)
	Standardized: β	0.03 (0.00–0.05)	0.09 (0.06–0.11)	0.09 (0.07–0.12)	0.13 (0.10–0.16)
	Q1 versus Q2–4: β	−0.04 (−0.09–0.01)	−0.16 (−0.22–−0.11)	−0.19 (−0.24–−0.13)	−0.26 (−0.32–−0.21)

AD: Alzheimer's disease; ADRD: Alzheimer's disease related dementias; HR: hazard ratio; CI: confidence interval; C-statistic: concordance statistic. Continuous represents betas per tenth point of the imaging marker. Standardized represents betas per standard deviation increase of the imaging marker.

Model 1: adjusted for age and sex

Model 2: adjusted for age, sex, and MRI markers (i.e., total brain volume, hippocampal volume [except hippocampal signature], white matter hyperintensities)

* Cognitive outcomes were modeled as standardized values.

Similar to the dementia analyses, we observed slightly stronger associations between the ADRD marker and cognition in women than in men, though interactions were non-significant (episodic memory: p_interaction _= 0.84; executive functioning: p_interaction _= 0.72, general cognitive function: p_interaction _= 0.29).

### Association with APOE ε4 carriership

We observed no significant associations between the presence of at least one *APOE*-ε4 allele and the ADRD signature, neither in the sample for dementia analyses (β 0.008 [ ± standard error (SE) 0.015], p-value 0.63), nor in the sample for analyses on cognitive assessment (β 0.007 [ ± SE 0.016], p-value 0.68).

## Discussion

In this large population-based cohort, we confirmed associations of the novel ADRD signature imaging marker with cognition and risk of dementia. However, in our head-to-head comparison of four neuroimaging markers, associations for the ADRD signature were not greater than for other AD-related imaging markers. Overall, hippocampal volume showed stronger associations with dementia and cognition than any of the cortical thickness measures.

Risk estimates of the ADRD signature for cognition and dementia were less profound in the Rotterdam Study cohort than in FHS and UCD-ADRC. This discrepancy could, in part, stem from differences in our study sample, comprising community-dwelling Europeans, compared to the U.S. population in the FHS and the clinic-based sample of the replication cohort at UCD-ADRC. Additionally, we used FreeSurfer rather than the DiReCT method employed by the FHS, which might contribute to the observed disparities due to differences in the underlying techniques between both approaches.^3^ However, our results for the Dickerson's signature and mean cortical thickness, which also rely on these methods, were similar compared to those in the FHS report. Notably, FreeSurfer is a widely used openly available neuroimaging analysis tool, and its application in our validation study probably reflects the performance of the ADRD signature if it were implemented in real-world populations.^18^

In the original study by Satizabal et al., the development of the ADRD signature was meticulously executed, including all necessary steps and external validation in a separate cohort.^3^ Despite these thorough efforts, our findings emphasize a critical reality, namely that results may not consistently replicate across different populations, due either to methodological differences or heterogeneity in the studied populations. The discrepancy between our findings and those from the FHS and UCD-ADRC cohort may however in fact also be explained by a third factor, namely chance. Results from one study will not always be replicated based on statistical chance alone. Replication studies are essential to establish robust biomarkers for dementia risk stratification, and external validation to determine in which setting they may or may not apply.

The current study is strengthened by its large, population representative sample of individuals with brain imaging and long-term follow-up for dementia. Some limitations need to be acknowledged. First, brain MRI was incorporated at the last stage (i.e., third day-visit) of each examination round of the Rotterdam Study, which might have led to selection of more healthy participants who underwent MRI. However, any selection would influence all four imaging markers equally and should not affect the head-to-head comparison. Second, the ADRD signature is developed using MRI data acquired with scanners different than ours and partly with different field strengths. Use of FreeSurfer rather than the DiReCT method might also have contributed to the observed disparities. Such methodological differences are important to consider, especially as translation to a real-world setting may depend on robustness of measures across different MRI scanners and algorithms.

In conclusion, in this population-based study the novel cortical thickness signature did not outperform hippocampal volume in the magnitude of the association with cognition and for dementia risk stratification. The importance of replication studies underlines the value of the current study. Replicating research findings is vital to establish robust biomarkers for dementia risk prediction.

## Supplemental Material

sj-docx-1-alz-10.1177_13872877251315044 - Supplemental material for Validation of a novel neuroimaging signature for dementia and clinical Alzheimer's disease in the population-based Rotterdam study Supplemental material, sj-docx-1-alz-10.1177_13872877251315044 for Validation of a novel neuroimaging signature for dementia and clinical Alzheimer's disease in the population-based Rotterdam study by Jacqueline J Claus, Mathijs T Rosbergen, Gennady V Roshchupkin, M Arfan Ikram, Meike W Vernooij and Frank J Wolters in Journal of Alzheimer's Disease
