# Supplementary material for: Validation of a novel neuroimaging signature for dementia and clinical Alzheimer's disease in the population-based Rotterdam study
Source: J Alzheimers Dis. 2025 Feb 16;108(1 Suppl):S316–23. doi: 10.1177/13872877251315044 (PMC12583652; doi:10.1177/13872877251315044)
Supplement: sj-docx-1-alz-10.1177_13872877251315044 - Supplemental material for Validation of a novel neuroimaging signature for dementia and clinical Alzheimer's disease in the population-based Rotterdam study [file sj-docx-1-alz-10.1177_13872877251315044.docx]

**Supplemental Material**

**Validation of a novel neuroimaging signature for dementia and clinical Alzheimer’s disease in the population-based Rotterdam Study**

**Supplemental Figure 1. Correlation between the neuroimaging signatures.** This figure displays the correlation matrix between four neuroimaging measures: the Alzheimer’s Disease Related Dementias (ADRD) signature, the Dickerson signature, mean overall cortical thickness, and hippocampal volume. Each cell in the matrix represents the Pearson correlation coefficient, ranging from -1 (strong negative correlation) to 1 (strong positive correlation). The diagonal elements of the matrix represent perfect correlations (correlation coefficient = 1) between the same measures. Off-diagonal elements show the correlations between different neuroimaging signatures, highlighting the relationships among these variables. The strength and direction of the correlations provide insight into how these neuroimaging markers are related in the context of neurodegenerative processes.

**
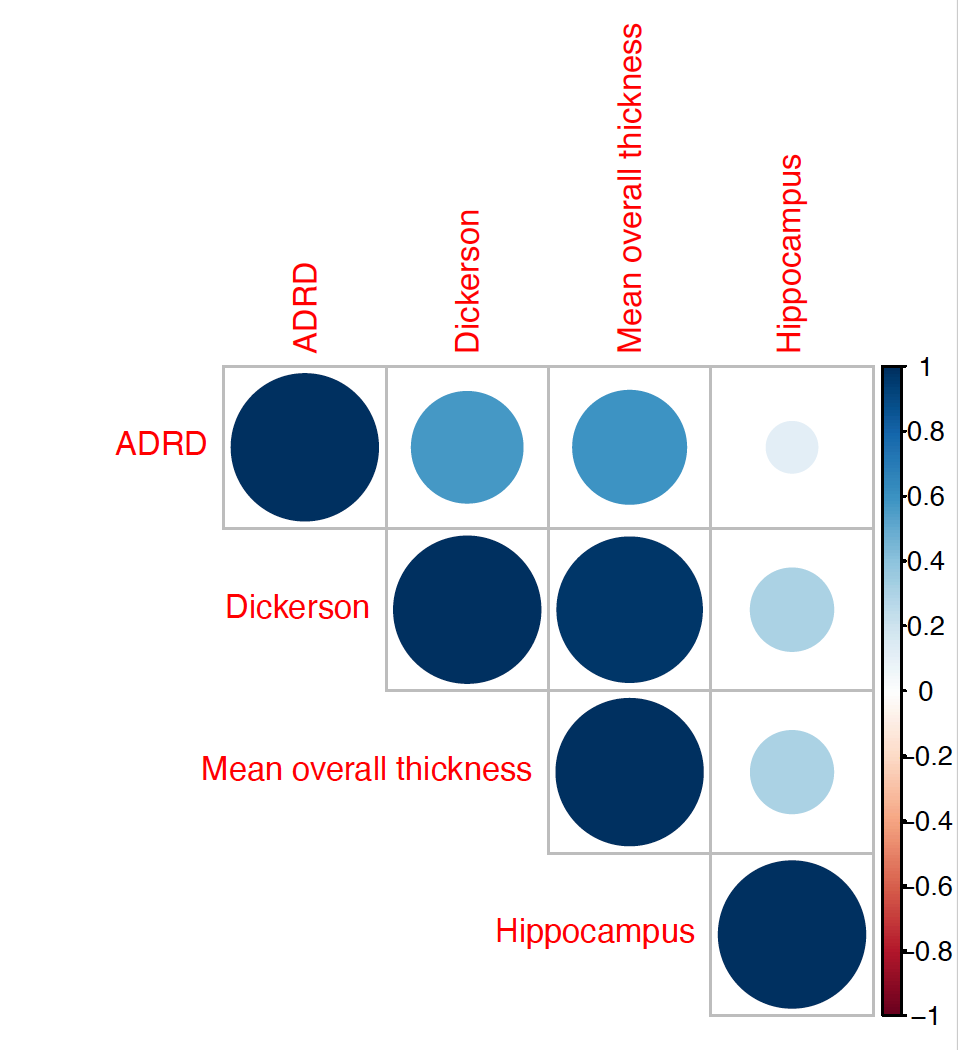
**
